# Incremental Value of MR Cholangiopancreatography in Diagnosis of Biliary Atresia

**DOI:** 10.1371/journal.pone.0158132

**Published:** 2016-06-24

**Authors:** Siyoun Sung, Tae Yeon Jeon, So-Young Yoo, Sook Min Hwang, Young Hun Choi, Woo Sun Kim, Yon Ho Choe, Ji Hye Kim

**Affiliations:** 1 Department of Radiology and Center for Imaging Science, Samsung Medical Center, Sungkyunkwan University School of Medicine, Seoul 06351, Korea; 2 Department of Radiology, Seoul National University Hospital, Seoul National University College of Medicine, Seoul 03080, Korea; 3 Department of Pediatrics, Samsung Medical Center, Sungkyunkwan University School of Medicine, Seoul 06531, Korea; Texas A&M University, UNITED STATES

## Abstract

**Purpose:**

To evaluate the incremental value of a combination of magnetic resonance cholangiopancreatography (MRCP) and ultrasonography (US), compared to US alone, for diagnosing biliary atresia (BA) in neonates and young infants with cholestasis.

**Materials and Methods:**

The institutional review board approved this retrospective study. The US and MRCP studies were both performed on 64 neonates and young infants with BA (n = 41) or without BA (non-BA) (n = 23). Two observers reviewed independently the US alone set and the combined US and MRCP set, and graded them using a five-point scale. Diagnostic performance was compared using pairwise comparison of the receiver operating characteristics (ROC) curve. The sensitivity, specificity, accuracy, positive predictive value (PPV), and negative predictive value were assessed.

**Results:**

The diagnostic performance (the area under the ROC curve [A_z_]) for diagnosing BA improved significantly after additional review of MRCP images; A_z_ improved from 0.688 to 0.901 (*P* = .015) for observer 1 and from 0.676 to 0.901 (*P* = .011) for observer 2. The accuracy of MRCP combined with US (observer 1, 95% [61/64]; observer 2 92% [59/64]) and PPV (observer 1, 95% [40/42]; observer 2 91% [40/44]) were significantly higher than those of US alone for both observers (accuracy: observer 1, 73% [47/64], *P* = 0.003; observer 2, 72% [46/64], *P* = 0.004; PPV: observer 1, 76% [35/46], *P* = 0.016; observer 2, 76% [34/45], *P* = 0.013). Interobserver agreement of confidence levels was good for US alone (ĸ = 0.658, *P* < .001) and was excellent for the combined set of US and MRCP (ĸ = 0.929, *P* < .001).

**Conclusion:**

Better diagnostic performance was achieved with the combination of US and MRCP than with US alone for the evaluation of BA in neonates and young infants with cholestasis.

## Introduction

Early diagnosis of biliary atresia (BA) is of great clinical importance because timely surgical intervention can restore bile flow and prevent worsening of liver disease [1]. Avoiding unnecessary laparotomy in patients with other causes of neonatal cholestasis is also essential, as this may contribute to morbidity and a considerable proportion of patients may not have BA [2, 3].

Many investigators have endeavored to distinguish BA from non-BA patients without the use of laparotomy. The preoperative diagnosis of BA using multiple ultrasonography (US) parameters has met with variable success [4–7]. A triangular cord sign, though helpful in the diagnosis, is not always present in every BA patient [4, 8]. Moreover, biochemical and histopathologic results may also overlap between BA and other causes of neonatal cholestasis [2]. Magnetic Resonance cholangiopancreatography (MRCP) is another useful and non-invasive examination for biliary disease, and offers visualization of the extrahepatic biliary tree, including the confluence of the right and left hepatic ducts. A literature review on the performance of MRCP for the diagnosis of BA indicates an accuracy of 71–100% [9–11] and a sensitivity of 90–100% [9, 11, 12]. Thus, no single radiologic investigation allows a reliable diagnosis to be made with certainty [3, 13]. A multidisciplinary approach is required to discriminate BA from non-BA in neonates and young infants.

The aim of this study was to evaluate the incremental value of a combination of MRCP and US, compared to US alone, for diagnosing BA in neonates and young infants with cholestasis.

## Materials and Methods

### Study population

This retrospective study was approved by Samsung Medical Center institutional review board, and the requirement for informed consent was waived. And patient information was anonymized and de-identified prior to analysis.

We searched our institutional database for both abdominal US examinations and MRCP performed between January 2000 to January 2016 using the search terms “BA,” “neonatal hepatitis,” “cholestasis,” “jaundice,” and “clay-colored stool,”. The search yielded 117 neonates and young infants. Of these, 51 patients with choledochal cysts and 5 patients with Alagille syndrome were excluded. Ultimately, 61 consecutive neonates and young infants were selected. Cases were defined as 41 patients (male to female ratio, 12:29) with BA confirmed with surgical cholangiography or histologic analysis of the biliary remnants (BA group). Twenty children were diagnosed as clinically suspected of having neonatal hepatitis, based on their clinical and laboratory improvement during the follow-up period (n = 19) or according to surgical cholangiography (n = 1). Another 3 patients diagnosed as neonatal hepatitis were collected from Seoul National University Hospital between 2010 and 2015. Eventually, 23 children (male to female ratio, 10:13) were included as a control (non-BA group).

The charts were reviewed for demographic information, clinical profiles, and laboratory data.

### Imaging techniques

All US examinations were performed by pediatric radiologists. The equipment used was US systems (Sequoia, Siemens Healthcare, Erlangen, German; LOGIQ E9, GE Healthcare, Waukesha, Wisconsin) with high resolution linear-array transducers (5–10 MHz, 9 MHz, and 6–15 MHz) and curved-array transducers (5–10 MHz and 6 MHz). All patients were fasted for at least 3 hours before US examination and none of the infants was sedated.

MR studies were performed by using a 1.5- or 3.0-T unit (Signa Advantage Horizon; GE Medical Systems, Milwaukee, WI, USA; Achieva, Philips Healthcare, Best, the Netherlands). The imaging protocol consisted of axial and coronal single shot spin echo T2-weighted images (TR/TE = 678-45958/69-140 ms, section thickness [ST] = 3–5 mm, field of view [FOV] = 140–280 mm, matrix = 150–400 x 160–393), non-breath hold, respiratory-triggered, heavily T2-weighted turbo spine echo images (1008-44082/150-958 ms, 3–5 mm, 160–280 mm, 148–256 x 76–256), and three-dimensional (3D) MRCP (1200-2100/650 ms, 2 mm, 280 mm, 140–256 x 130–170).The 3D acquisition images were reformatted using a standard maximum intensity projection algorithm to create a rotating display. The MR examinations were performed during natural sleep, if possible. If this was judged impossible, sedatives were offered by a skilled pediatric sedation team, with parental consent.

### Image analysis

Two pediatric radiologists, who were blinded to the final clinical diagnosis, reviewed the two image sets (the US alone set and the combined US and MRCP set) parted by an interval of one month.

The parameters assessed by US included the length of the gallbladder (GB) along the longitudinal axis, with a description of its wall regularity. The GB was considered atretic when it was not visualized or when it was less than 1.9 cm long [4, 5]. Positivity for the triangular cord sign was defined as a thickness of the echogenic anterior to the right portal vein greater than 4 mm [14]. The caliber of the hepatic artery was measured at the level of the right proximal hepatic artery running parallel to the right portal vein. Hepatic artery enlargement was defined as a caliber of the hepatic artery greater than 1.5 mm [6]. The visibility of the common bile duct at the porta hepatis was recorded by US [4]. The visibility of the extrahepatic biliary tree was evaluated by MRCP. Our use of the term “extrahepatic biliary tree” refers to the common hepatic duct and the common bile duct, as well as the confluence of the right and left hepatic ducts [11].

Two observers first independently interpreted the US images alone, and they rated their confidence levels according to BA versus non-BA using a five-point scale (1 = definitely non-BA, 2 = probably non-BA, 3 = equivocal BA, 4 = probably BA, 5 = definite BA). The two endpoints of the five-point confidence level scale were predefined. The GB abnormalities (either atretic GB or GB wall irregularity), the presence of the triangular cord sign, hepatic artery enlargement, and non-visualized common bile duct at the porta hepatis were regarded as definite US findings of BA. Conversely, definitive US findings of non-BA were the absence of GB abnormalities (a normal length of the GB and GB wall regularity), absence of a triangular cord sign, absence of hepatic artery enlargement, and visualization of the common bile duct at the porta hepatis.

After one month later, two observers independently interpreted the combined set of US and MRCP images and graded their confidence levels using the same five-point scale. The diagnosis of BA was assigned according to the non-visualization of the extrahepatic biliary tree by MRCP. A study finding was considered to be non-BA when the entire extrahepatic biliary tree was visualized. When the MRCP diagnosis differed from that seen on US images, the observers were requested to place priority to the MRCP data.

### Statistical analysis

Statistical analyses were performed using SPSS software (SPSS, version 23.0; SPSS, Chicago, Ill). Results were considered significant if the *P*-value was less than 0.05. Descriptive data were expressed as the mean ± standard deviation (SD) or frequencies. Statistical differences were tested by applying a two-sample t-test, the Mann-Whitney test, or Fisher’s exact test. The diagnostic performance of two observers was compared by receiver operating characteristic (ROC) analysis with the area under the ROC curve (A_z_) and pairwise comparison. The sensitivity, specificity, accuracy, and positive and negative predictive values of each observer were calculated. McNemar’s test was used to compare the sensitivity, specificity, and accuracy, and Bennett’s test was used to compare the positive and negative predictive values of each observer [15].

## Results

### Clinical profiles and laboratory data

The demographic information and laboratory data for patients with BA and non-BA are summarized in Table 1. The mean interval between US and MRCP was 3.5 days (range, 0–30 days) in BA patients. All patients underwent Kasai portoenterostomy (mean age 81 days, range 32 days– 7 months). In non-BA patients, the mean interval between US and MRCP was 2.9 days (range, 0–19 days). The BA patients showed a female predominance. No statistically significant differences were noted in the frequency of clay-colored stool or in the mean levels of biochemical hepatic function tests in both groups.

**Table 1:** Clinical profile and laboratory data.

Variables	BA (n = 41)	Non-BA (n = 23)	*P*
Gender, male: female^**a**^	12: 29	10: 13	0.382
Age at US, days^**b**^	73 ± 41	62 ± 38	0.295
Clay stool^**a**^	30/41 (73%)	11/23 (48%)	0.079
Total bilirubin	10.6 ± 3.2	11.1 ± 5.4	0.643
Direct bilirubin	7.5 ± 2.4	7.5 ± 4.0	1.000
AST	257 ± 187	202 ± 181	0.258
ALT	177 ± 151	155 ± 162	0.588
ALP	755 ± 392	645 ± 320	0.256

AST, aspartate aminotransferase; ALT, alanine aminotransferase; ALP, alkaline phosphatase.

^a^Data are numbers of patients.

^b^Data are median ± standard deviation.

Unless otherwise noted, data are means ± standard deviation.

### US and MRCP imaging findings

The US and MRCP findings in both the BA and non-BA patients are summarized in Table 2. The US findings of GB abnormalities (observer 1, *P*<0.001; observer 2, *P*<0.001) and hepatic artery enlargement (observer 1, *P* = 0.002; observer 2, *P*<0.001) were significantly higher in the BA patients than in the non-BA patients for both observers. The GB was visualized in 58 patients (BA, n = 36; non-BA, n = 22) and was not seen in 6 patients (BA, n = 5; non-BA, n = 1). The proportions of triangular cord sign and non-visualization of the common bile duct were not significantly different between the BA and non-BA patients, as determined by both observers. Non-visualization of the extrahepatic biliary tree by MRCP was significantly higher in the BA patients than in the non-BA patients (observer 1, *P*<0.001; observer 2, *P*<0.001).

**Table 2:** US and MRCP findings in patients with BA and non-BA.

	Observer 1			Observer 2		
	BA	Non-BA	*P*	BA	Non-BA	*P*
US findings						
GB abnormalities	90 (37/41)	39 (9/23)	< 0.001	93 (38/41)	48 (11/23)	< 0.001
Atretic GB	73 (30/41)	35 (8/23)	0.006	63 (26/41)	43 (10/23)	0.201
GB wall irregularity	75 (27/36)	27 (6/22)	0.001	67 (24/36)	32 (7/22)	0.021
Triangular cord sign	56 (23/41)	30 (7/23)	0.087	54 (22/41)	26 (6/23)	0.061
Hepatic artery enlargement	73 (30/41)	30 (7/23)	0.002	78 (32/41)	26 (6/23)	< 0.001
Absence of CBD	92 (12/13)	53 (8/15)	0.063	80 (12/15)	44 (7/16)	0.089
MRCP finding						
Absence of EBT	98 (40/41)	9 (2/23)	< 0.001	98 (40/41)	17 (4/23)	< 0.001

GB, gallbladder; CBD, common bile duct; EBT, extrahepatic biliary tree. Data are percentage (numbers of patients/total).

### Incremental value of MRCP for diagnostic performance

**Fig 1:**
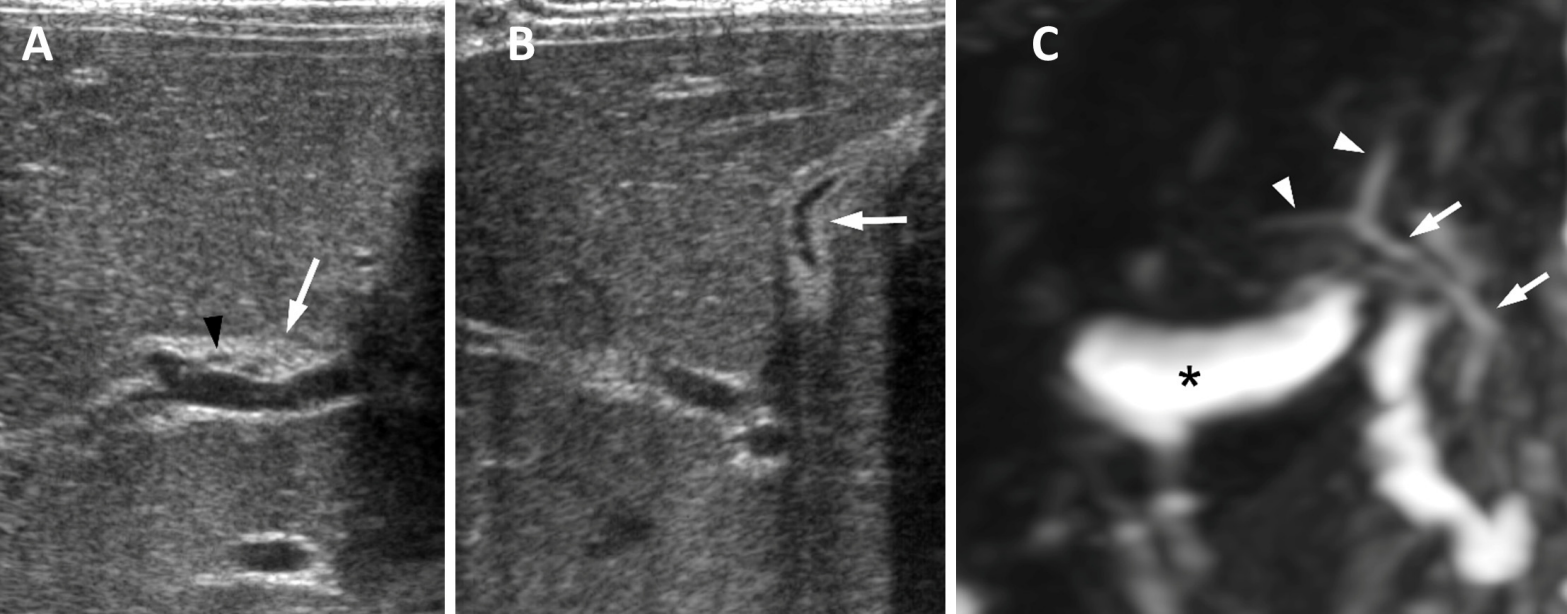
Neonatal hepatitis in a 54-day-old girl. (A) US image in transverse plane shows echogenic thickening anterior to the right portal vein measuring 4.2 mm in thickness (positive triangular cord sign) (arrow). Diameter of right hepatic artery is 0.7 mm (arrowhead). (B) US image in oblique subcostal plane shows atretic gallbladder measuring 0.9 cm (arrowhead). US confidence level using a five-point scale is 3 (equivocal biliary atresia) by both observers. (C) 3D MRCP image shows the common hepatic and common bile ducts (arrows), the confluence of the right and left hepatic ducts (arrowheads), and normal gallbladder (asterisk). Non-biliary atresia was diagnosed correctly by both observers after additional review of MRCP images.

**Fig 2:**
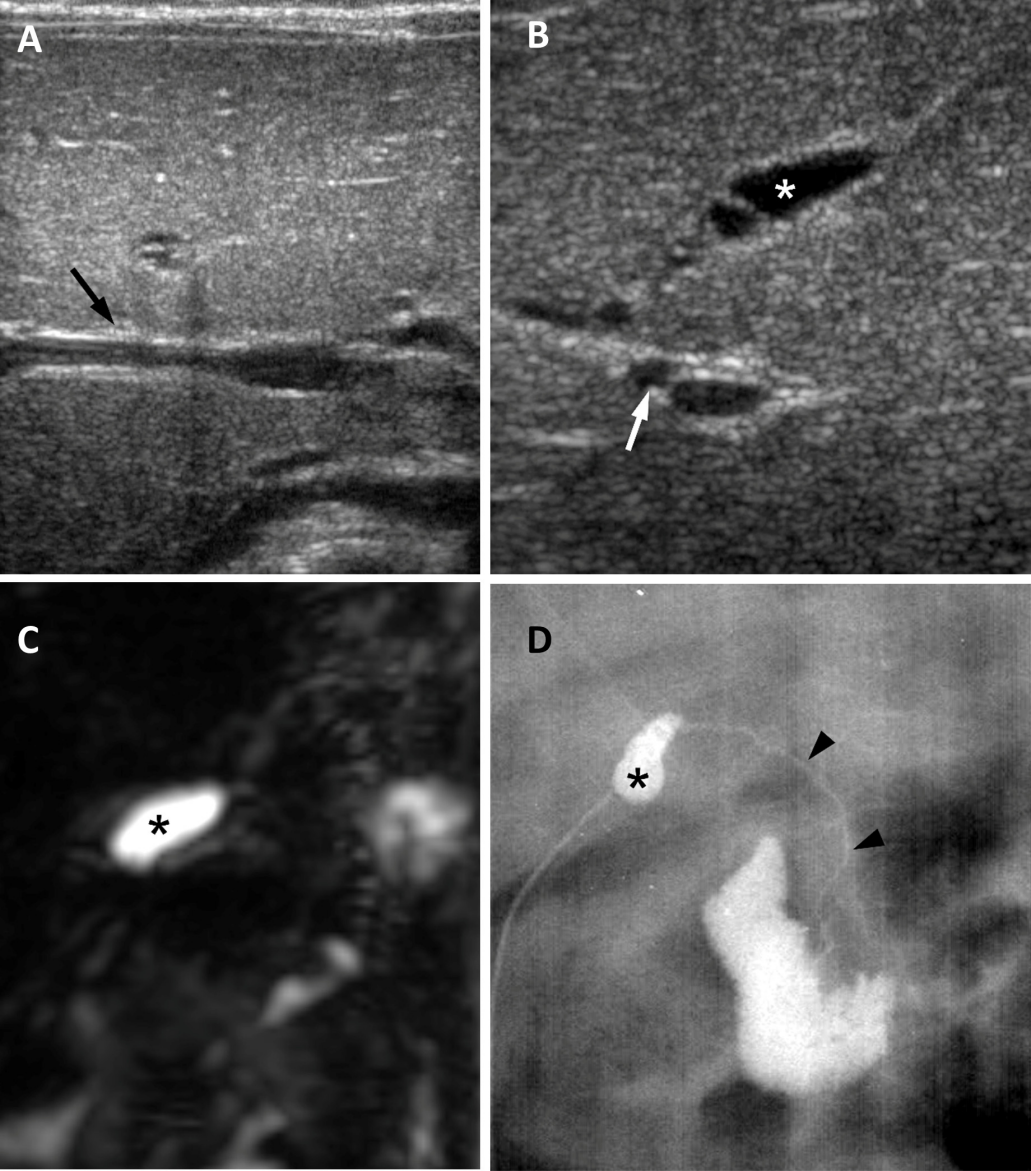
Biliary atresia in a 65-day-old girl. (A) US image in transverse plane shows negative triangular cord sign (arrow). (B) US image in oblique subcostal plane shows atretic gallbladder measuring 0.8 cm (asterisk) and enlarged hepatic artery measuring 1.5 mm (arrow). US confidence level using a five-point scale is 3 (equivocal biliary atresia) by both observers. (C) 3D MRCP image shows no visible extrahepatic biliary tree and small gallbladder (asterisk). Biliary atresia was diagnosed with certainty by both observers after additional review of MRCP images. (D) Surgical cholangiography shows small gallbladder (asterisk) and a patent but extremely hypoplastic common bile duct (arrowheads), suggesting type 2 biliary atresia.

The diagnostic performance with respect to evaluation of BA by two observers increased significantly after additional review of the MRCP images: A_z_ increased from 0.688 to 0.901 (*P* = 0.015) for observer 1 and from 0.676 to 0.901 for observer 2 (*P* = 0.011) (Table 3). Table 3 demonstrates the diagnostic predictive values to diagnose non-BA for each observer and by each technique. The confidence levels for interobserver agreement were good for US alone (ĸ = 0.658) and excellent for the combined set of US and MRCP (ĸ = 0.929). The diagnostic accuracy (observer 1, *P* = 0.003; observer 2, *P* = 0.004) and positive predictive value (observer 1, *P* = 0.016; observer 2, *P* = 0.013) were significantly higher for both observers when both US and MRCP images were reviewed than when US images alone were reviewed. The sensitivity was higher when analyzing both US and MRCP images than when analyzing US images alone, but the differences did not reach statistical significance in both observers. The specificity and negative predictive value demonstrated the tendency of improvement after additional review of the MRCP images, but statistical significance was different in each observer. Additional review of the MRCP data permitted the observers to correct some diagnostic errors occurring with US images only (observer 1, n = 17; observer 2, n = 16) (Table 4) (Figs 1 and 2). Five cases (BA, n = 1; non-BA, n = 4) were not correctly interpreted by either observer even after additional review of the MRCP findings.

**Table 3:** Comparison of diagnostic performance of US alone and US with MRCP for discrimination between BA and non-BA.

	Type of imaging		*P*
	US alone	US with MRCP	
Observer 1			
A_z_	0.688	0.901	0.015
Senstivity	85 (35/41)	98 (40/41)	0.125
Specificity	52 (12/23)	91 (21/23)	0.023
Accuracy	73 (47/64)	95 (61/64)	0.003
Positive predictive value	76 (35/46)	95 (40/42)	0.016
Negative predictive value	67 (12/18)	95 (21/22)	0.052
Observer 2			
A_z_	0.676	0.901	0.011
Senstivity	83 (34/41)	98 (40/41)	0.070
Specificity	52 (12/23)	83 (19/23)	0.215
Accuracy	72 (46/64)	92 (59/64)	0.004
Positive predictive value	76 (34/45)	91 (40/44)	0.013
Negative predictive value	63 (12/19)	95(19/20)	0.039

A_z_, the area under the receiver operating characteristic curve. Except A_z_, data are percentages with numbers of patients in parentheses.

**Table 4:** Interobserver cross-classification of results before and after MRCP interpretation.

	Correct before MRCP interpretation	Incorrect before MRCP interpretation
Observer 1		
Correct after MRCP interpretation	44	17
BA	34	6
Non-BA	10	11
Incorrect after MRCP interpretation	3	0
BA	1	0
Non-BA	2	0
Observer 2		
Correct after MRCP interpretation	43	16
BA	33	7
Non-BA	10	9
Incorrect after MRCP interpretation	3	2
BA	1	0
Non-BA	2	2
Both observer 1 and observer 2		
Correct after MRCP interpretation	40	12
BA	31	4
Non-BA	9	8
Incorrect after MRCP interpretation	2	0
BA	1^**a**^	0
Non-BA	1^**b**^	0

Data are numbers of patients.

^a^ In this BA patient, US confidence level was 3 (equivocal BA), whereas MRCP made a misdiagnosis as non-BA in both observers because the high signals of the bowels masqueraded as patent extrahepatic biliary tree.

^b^ In this non-BA patient, US confidence level was 2 (probably non-BA), whereas MRCP made a misdiagnosis as BA in both observers due to small diameter of the extrahepatic biliary tree.

## Discussion

We evaluated the added value of MRCP to US images for distinguishing BA from non-BA in neonates and young infants with cholestasis. Our results showed that the A_z_, diagnostic accuracy, and positive predictive value significantly increased for both observers when both MRCP and US images were reviewed, compared with US images alone. In addition, adding MRCP to US images decreased the diagnostic errors by both observers.

The US features, such as GB abnormalities, triangular cord sign, and vascular alterations, have been reported as significant predictors for discriminating BA from non-BA. Variable ranges of sensitivity and specificity were reported in the literature for individual US parameters, such as abnormal GB (61–97% and 69–100%) [3–5, 7, 16], triangular cord sign (23–100% and 87–100%) [3, 4, 7, 16, 17], enlarged hepatic artery (72–92% and 49–87%) [4, 6, 18], and non-visualized common bile duct (83–95% and 48–92%) [4, 6, 7, 19]. Humphrey and Stringer described that the use of a combination of several US parameters allowed the distinguishing of BA from other causes of neonatal cholestasis, with an overall accuracy of 98% [7]. In contrast to this high diagnostic performance of US, Giannattasio et al. [8] found that 68% (17/25) of infants with BA showed an identifiable GB at US, with a regular wall in one-fifth of the infants, and the triangular cord sign was seen in 24% (6/25) of cases. The lower sensitivity of US for the diagnosis of BA could reflect several potential pitfalls of US diagnosis itself (such as detection of a small non-distended GB and the difficulty in visualization of the common bile duct in healthy infants) or obscured triangular cord sign in patients with early-stage BA [4, 8].

Therefore, the assessment of BA is challenging, and no single radiologic examination appears to be clearly superior, although several efforts have attempted to improve the preoperative investigation for BA. A BA diagnostic scoring system consisted of clinical, laboratory, and histopathological variables, as well as US parameters [3], has recently been developed. Subsequent validation in 75 consecutive patients revealed an overall high diagnostic accuracy of 99% (74/75) [3]. However, the indications for liver biopsy were debated by Sciveres et al. [20] based on their experience. When liver biopsy was proposed in only 25% (16/64), they found a similar diagnostic performance (three diagnostic errors out of 64 cases) to that of new scoring system introduced by El-Guindi et al. [3].

MRCP can be a useful and non-invasive method for the evaluation of hepatobiliary disease, even in pediatric patients. Previous studies have reported that BA can be reliably diagnosed by visualization of the extrahepatic biliary tree, especially when accompanied by the appearance of an atrophic GB by MRCP [9–12]. Jaw et al. [10] reported that 16 jaundiced infants were correctly diagnosed as BA (n = 6) or non-BA (n = 10) with an overall accuracy of 100%, based on non-visualization of the extrahepatic biliary tree on MRCP. However, Norton et al. [11] reported that their three false positive cases indicated that the previous criteria established by Jaw et al. [10] could not be relied on alone for a 100% diagnostic accuracy. They found that MRCP was 82% (19/23) accurate in depicting BA when using their expanded criteria, which included delineation of the confluence, common hepatic, and common bile ducts, in agreement with our criteria used in the present study.

Both observers in our study made erroneous diagnoses of BA in five patients, even after additional review of the MRCP images. Among these errors, a false positive diagnosis of BA was made in four patients. In one infant, the extrahepatic biliary tree, including the confluence, was not visualized by MRCP. In the other three infants, the common hepatic and common bile ducts were observed, but the confluence was not visualized clearly. These cases signify a potential pitfall in the diagnosis of BA by MRCP, as it depends on the inadequate production and secretion of bile. A prospective pilot study by Siles et al. [21] reported that MRCP visualization of the normal biliary system was only possible in 62.5% (10/16) of normal infants younger than three months. The MRCP findings may be simulating BA when insufficient bile is produced in the extremely small diameter of the hypoplastic bile duct [9, 11]. Except in one case, the delineation of the extrahepatic biliary tree that included confluence ruled out the possibility of BA, with a negative predictive value of 95% (21/22) for observer 1 and 95% (19/20) for observer 2. One false negative case was diagnosed as having type II BA; in this patient, the common bile duct was preserved, but the common hepatic duct and confluence were not visualized by surgical cholangiography. The high signal intensity by intestinal tracts may masquerade as normal extrahepatic biliary tree.

Our study has several drawbacks. First, although we recruited consecutive patients who met our inclusion criteria, the possibility of selection bias should be considered due to the retrospective study design. Second, the increased diagnostic accuracy observed for the combined image sets may be related to the reviewers’ experiences with the rating system. A recall bias may have resulted from the order of image evaluation, although we tried to prevent this issue by using a one-month interval for review and by rearranging the order of the cases. Third, our number of non-BA patients was small and our statistical results could be affected by the low number of non-BA patients. A large-scale research may be needed to validate the value of MRCP for differentiating BA from non-BA in neonates and young infants.

In conclusion, the addition of MRCP yields better diagnostic accuracy and positive predictive value than the use of US alone in the diagnosis of BA in newborn and young infants with cholestasis. Therefore, when US is inconclusive in differentiating BA from non-BA, additional MRCP imaging may provide valuable information regarding the normal patency of the extrahepatic biliary tree and can reduce or even obviate unnecessary laparotomy.

## References

[1] Hartley JL, Davenport M, Kelly DA. Biliary atresia. Lancet. 2009;374: 1704–1713. 10.1016/S0140-6736(09)60946-6 19914515

[2] Rastogi A, Krishnani N, Yachha SK, Khanna V, Poddar U, Lal R. Histopathological features and accuracy for diagnosing biliary atresia by prelaparotomy liver biopsy in developing countries. J Gastroenterol Hepatol. 2009;24: 97–102. 10.1111/j.1440-1746.2008.05737.x 19196397

[3] El-Guindi MA, Sira MM, Sira AM, Salem TA, El-Abd OL, Konsowa HA, et al Design and validation of a diagnostic score for biliary atresia. J Hepatol. 2014;61: 116–123. 10.1016/j.jhep.2014.03.016 24657403

[4] Mittal V, Saxena AK, Sodhi KS, Thapa BR, Rao KL, Das A, et al Role of abdominal sonography in the preoperative diagnosis of extrahepatic biliary atresia in infants younger than 90 days. AJR Am J Roentgenol. 2011;196: W438–445. 10.2214/AJR.10.5180 21427309

[5] Tan Kendrick AP, Phua KB, Ooi BC, Tan CE. Biliary atresia: making the diagnosis by the gallbladder ghost triad. Pediatr Radiol. 2003;33: 311–315. 12695863 10.1007/s00247-003-0867-z

[6] Kim WS, Cheon JE, Youn BJ, Yoo SY, Kim WY, Kim IO, et al Hepatic arterial diameter measured with US: adjunct for US diagnosis of biliary atresia. Radiology. 2007;245: 549–555. 17890351 10.1148/radiol.2452061093

[7] Humphrey TM, Stringer MD. Biliary atresia: US diagnosis. Radiology. 2007;244: 845–851. 17709832 10.1148/radiol.2443061051

[8] Giannattasio A, Cirillo F, Liccardo D, Russo M, Vallone G, Iorio R. Diagnostic role of US for biliary atresia. Radiology. 2008;247: 912; author reply 912–913. 10.1148/radiol.2473071715 18487545

[9] Liu B, Cai J, Xu Y, Peng X, Zheng H, Huang K, et al Three-dimensional magnetic resonance cholangiopancreatography for the diagnosis of biliary atresia in infants and neonates. PLoS One. 2014;9: e88268 10.1371/journal.pone.0088268 24505457 PMC3914942

[10] Jaw TS, Kuo YT, Liu GC, Chen SH, Wang CK. MR cholangiography in the evaluation of neonatal cholestasis. Radiology. 1999;212: 249–256. 10405749 10.1148/radiology.212.1.r99jl13249

[11] Norton KI, Glass RB, Kogan D, Lee JS, Emre S, Shneider BL. MR cholangiography in the evaluation of neonatal cholestasis: initial results. Radiology. 2002;222: 687–691. 11867786 10.1148/radiol.2223010969

[12] Han SJ, Kim MJ, Han A, Chung KS, Yoon CS, Kim D, et al Magnetic resonance cholangiography for the diagnosis of biliary atresia. J Pediatr Surg. 2002;37: 599–604. 11912518 10.1053/jpsu.2002.31617

[13] Moyer V, Freese DK, Whitington PF, Olson AD, Brewer F, Colletti RB, et al Guideline for the evaluation of cholestatic jaundice in infants: recommendations of the North American Society for Pediatric Gastroenterology, Hepatology and Nutrition. J Pediatr Gastroenterol Nutr. 2004;39: 115–128. 15269615 10.1097/00005176-200408000-00001

[14] Lee HJ, Lee SM, Park WH, Choi SO. Objective criteria of triangular cord sign in biliary atresia on US scans. Radiology. 2003;229: 395–400. 14595143 10.1148/radiol.292020472

[15] Bennett BM. On comparisons of sensitivity, specificity and predictive value of a number of diagnostic procedures. Biometrics. 1972;28: 793–800. 5073252

[16] Kanegawa K, Akasaka Y, Kitamura E, Nishiyama S, Muraji T, Nishijima E, et al Sonographic diagnosis of biliary atresia in pediatric patients using the "triangular cord" sign versus gallbladder length and contraction. AJR Am J Roentgenol. 2003;181: 1387–1390. 14573442 10.2214/ajr.181.5.1811387

[17] Kotb MA, Kotb A, Sheba MF, El Koofy NM, El-Karaksy HM, Abdel-Kahlik MK, et al Evaluation of the triangular cord sign in the diagnosis of biliary atresia. Pediatrics. 2001;108: 416–420. 11483808 10.1542/peds.108.2.416

[18] Zhou LY, Wang W, Shan QY, Liu BX, Zheng YL, Xu ZF, et al Optimizing the US Diagnosis of Biliary Atresia with a Modified Triangular Cord Thickness and Gallbladder Classification. Radiology. 2015;277: 181–191. 10.1148/radiol.2015142309 25955579

[19] Azuma T, Nakamura T, Nakahira M, Harumoto K, Nakaoka T, Moriuchi T. Pre-operative ultrasonographic diagnosis of biliary atresia—with reference to the presence or absence of the extrahepatic bile duct. Pediatr Surg Int. 2003;19: 475–477. 12750934 10.1007/s00383-003-0962-0

[20] Sciveres M, Milazzo MP, Maggiore G. A scoring system for biliary atresia: is this the right one? J Hepatol. 2015;62: 985–986. 10.1016/j.jhep.2014.11.042 25500723

[21] Siles P, Aschero A, Gorincour G, Bourliere-Najean B, Roquelaure B, Delarue A, et al A prospective pilot study: can the biliary tree be visualized in children younger than 3 months on Magnetic Resonance Cholangiopancreatography? Pediatr Radiol. 2014;44: 1077–1084. 10.1007/s00247-014-2953-9 24710862

